# Rechallenge with Amisulpride in a Patient with Schizophrenia following a Manic Episode during Previous Therapy

**DOI:** 10.1155/2022/8732708

**Published:** 2022-05-16

**Authors:** Li-Yu Hu, Chen-Jee Hong, Shih-Jen Tsai, Cheng-Che Shen

**Affiliations:** ^1^Department of Psychiatry, Kaohsiung Veterans General Hospital, Taiwan; ^2^School of Medicine, National Yang-Ming University, Taipei, Taiwan; ^3^Department of Psychiatry, Taipei Veterans General Hospital, Taipei, Taiwan; ^4^Institute of Brain Science, National Yang-Ming University, Taipei, Taiwan; ^5^Department of Psychiatry, Chiayi Branch, Taichung Veterans General Hospital, Chiayi, Taiwan; ^6^Department of Information Management, National Chung-Cheng University, Chiayi, Taiwan

## Abstract

Amisulpride is an atypical antipsychotic. It is also effective in treating depression. The potential antidepressant effect raises the concern that amisulpride can induce mania. However, reports of amisulpride-induced mania have been rare. Here, we present the case of a Taiwanese woman with a 22-year history of schizophrenia. At the age 57 years, she developed manic symptoms while on treatment with amisulpride for six weeks. She was immediately admitted to the psychiatric in-patient unit. The manic symptoms completely subsided within eight days without the administration of any mood stabilizer. Readministration of a single dose of 200 mg amisulpride during hospitalization induced the same manic symptoms, which remitted completely within 24 hours without any mood stabilizer administration.

## 1. Introduction

Amisulpride is a benzamide derivative initially developed as a selective D2/D3 receptor antagonist for treating schizophrenia [1]. In addition, amisulpride has a unique dose-related pharmacological feature, which lets the medication have more clinical applications at different dosages. At higher doses, amisulpride blocks postsynaptic dopamine D2 and D3 receptors in the limbic and prefrontal regions, responsible for its antipsychotic effect. However, at low doses, it blocks presynaptic autoreceptors, which facilitate the dopamine release and thus may resolve the negative symptoms caused by dopaminergic hypoactivity and the symptoms of depression [2]. In short, it is effective in treating depression [3, 4]. The potential antidepressant effect raises the concern that amisulpride can induce mania. We could identify only five case reports related to amisulpride-induced mania on MEDLINE [5–9]. However, in most of these cases, medications such as antidepressants were administered concomitantly, and comorbidity with certain cerebral diseases, such as epilepsy, was observed. Furthermore, a rechallenge with amisulpride was not attempted. Therefore, causality could not be ascertained. Here, we present the case of a Taiwanese woman who was 57 years old in 2009. She was diagnosed with schizophrenia in 1987. She was being treated with amisulpride since April 2009, and manic symptoms developed on May 23, 2009. She was immediately admitted to the psychiatric in-patient unit of Taipei Veterans General Hospital. The manic symptoms were completely remitted within eight days without administering any mood stabilizer. Readministration of a single dose of 200 mg of amisulpride during hospitalization induced similar manic symptoms, which remitted completely within 24 hours without mood stabilizer administration.

## 2. Case Presentation

In 1987, Mrs. Xu, a 35-year-old woman with high school education, unemployed, married with two boys and one girl, living with her husband and all children, with no habit of drinking alcohol or taking any other illicit substance, was diagnosed with schizophrenia. She was introvert and polite before the onset of schizophrenia. There was no previous physical or mental illness history confirmed by her family. Her clinical manifestations included auditory hallucinations, persecutory delusion, and misidentification of persons. She was admitted to psychiatric wards on more than ten occasions, primarily due to exacerbations of psychotic positive symptoms. However, even if the positive symptoms were stable, at home, due to progressively developed negative symptoms, it was gradually challenging for the patient to play the role of a competent housewife. Drug compliance was not good because of the unbearable adverse effects of antipsychotics, including risperidone-related hyperprolactinemia, olanzapine-related body weight gain, and clozapine-induced sialorrhea. Hence, she has been maintained on 30–40 mg of haloperidol per day with an unsatisfactory treatment effect. The patient was depressed and tearful when disturbed by the delusions or hallucinations. However, she was able to maintain a sheltered job for 13 years. To improve her persistent psychotic symptoms further, haloperidol was replaced with amisulpride at 600 mg/day on April 10, 2009. One week later, the patient reported reduced hallucinations and delusions. Three weeks into amisulpride use, her psychotic symptoms nearly resolved, and she was discharged. The patient was happy to be considered appropriate, and her family was satisfied with her condition on amisulpride treatment. However, two months after the initiation of amisulpride, the patient unexpectedly experienced a sudden manic episode with agitation, elated mood, hypertalkativeness, hyperactivity, grandiose delusion, and violence. She was admitted to the hospital with a working diagnosis of amisulpride-induced mania. During the initial five days of hospitalization, all antipsychotics were withdrawn. The patient's manic symptoms persisted and required occasional temporary physical restraints to prevent injury to herself and others. Chlorpromazine at 400 mg/day was used to sedate the patient on day 6. The patient returned to her clinical baseline with prominent hallucinations, delusions, and secondary depression on day 8. On day 15, at the request of the patient's daughter, 200 mg of amisulpride was readministered and the dosage of chlorpromazine was reduced to 300 mg/day. The patient and her daughter were informed of the mania reemergence risk, and the patient signed her consent. Approximately 3–4 hours after the administration of amisulpride, the patient started to display manic symptoms, such as elated mood, hyperactivity, grandiosity, and pressured speech. Amisulpride was discontinued, and chlorpromazine was continued at 300 mg/day, and the patient's manic symptoms resolved within 24 hours. Eventually, the patient's psychotic symptoms were controlled with the administration of haloperidol at 40 mg/day and the discontinuation of chlorpromazine and amisulpride.

After 2009, the patient was admitted to the acute psychiatric ward three times in our hospital due to similar psychotic exacerbations. During her last admission in 2014, we discontinued the haloperidol 40 mg/day and prescribed clozapine 300 mg/day for the refractory psychotic symptoms. After that, her family sent her to a psychiatric ward for long-term treatment in another hospital, where she has been treated until this report was written. We traced and scrutinized the patient's medical records since 2009. The patient was continued on clozapine 300 mg/day since 2014, and there was no evidence of any new manic episode in the last twelve years.

## 3. Discussion

Although various hypotheses have been proposed, the mechanism of antipsychotic-induced mania remains unknown. Several studies have suggested that atypical antipsychotic-induced mania may be associated with frontal dopamine release through serotonin 5-HT_2a_ receptor blockade [10]. By contrast, the presumed selectivity of amisulpride for D2 and D3 dopamine receptors has led to the prevailing hypothesis that modulation of dopaminergic signaling is responsible for the antidepressant efficacy. However, no D2/D3 antagonist other than amisulpride has proved effective in animal or human studies [11]. Abbas et al. demonstrated that amisulpride is a potent human 5-HT_7a_ receptor antagonist essential for antidepressant activity [12].

We could identify five case reports of amisulpride-induced mania in the literature [5–9]. However, in most of these cases, patients received other medications which might have induced concomitant mania, and comorbidity with certain cerebral diseases, such as epilepsy, was observed. For example, in the case report by Kim et al. [4], the patient was administered citalopram 20 mg/day, and the possibility of the manic episode being induced by citalopram cannot be precluded. In the present case, mania was induced by the administration of a single dose of amisulpride 200 mg and resolved within 24 hours without the administration of an antidepressant or mood stabilizer. Thus, the mania can be rationally assumed to have been induced by the administration of amisulpride 200 mg rather than a reduction in the chlorpromazine dose, because the manic episode occurred and was resolved while the patient was maintained on the same chlorpromazine dose. We conclude that the manic episode, in this case, was induced by amisulpride.

Based on our report and the literature, special consideration should be given to the possibility of amisulpride-induced mania among patients with schizophrenia treated with concomitant antidepressants and amisulpride, with low-dose amisulpride for negative symptoms or depressive symptoms, as well as amisulpride-treated patients with epilepsy and any cerebral disease, like cerebral palsy. In addition, a tentative diagnosis of schizoaffective disorder should be evaluated as a possible partial explanation for the emerging amisulpride-associated mania.

## Data Availability

The data that support the findings of this study are not available due to patients' privacy. Interested individuals should contact the Taipei Veterans General Hospital to gain access.
